# Awareness of Repetitive Strain Injury of the Fifth Finger and Its Association With Fifth Finger Pain Among Young Adults Due to Overuse of Smartphones

**DOI:** 10.7759/cureus.114186

**Published:** 2026-08-08

**Authors:** Rohan R Gadave, Pragati R Patil

**Affiliations:** 1 Department of Physiotherapy, Krishna College of Physiotherapy, Krishna Vishwa Vidyapeeth (Deemed to be University), Karad, IND; 2 Department of Neuro-Physiotherapy, Krishna College of Physiotherapy, Krishna Vishwa Vidyapeeth (Deemed to be University), Karad, IND

**Keywords:** awareness, ergonomics, fifth finger pain, musculoskeletal disorders, repetitive strain injury, smartphone overuse, young adults

## Abstract

Introduction

The widespread use of smartphones has substantially increased among young adults because of academic, occupational, and social demands. Prolonged smartphone use, particularly during texting, scrolling, gaming, and other repetitive hand activities, has been associated with musculoskeletal symptoms affecting the hand and fingers. The fifth finger plays a significant role in supporting handheld devices during one-handed smartphone use and may be subjected to sustained mechanical loading, potentially contributing to pain, discomfort, muscle fatigue, and repetitive strain symptoms. Despite increasing smartphone dependence, awareness of repetitive strain injury (RSI) involving the fifth finger and its relationship with fifth finger pain remains limited. Assessing this awareness and its association with smartphone use may help inform preventive strategies and promote ergonomic smartphone practices among young adults.

Methods

A cross-sectional, questionnaire-based study was conducted among 188 young adults to evaluate awareness of RSI of the fifth finger and its association with fifth finger pain related to smartphone overuse. Participants voluntarily completed a structured Google Forms questionnaire that collected information regarding smartphone usage duration, smartphone weight, hand-holding patterns, awareness of RSI, and symptoms involving the fifth finger. Descriptive statistical analysis was performed to summarize participant responses and evaluate the association between smartphone use characteristics and fifth finger pain.

Conclusion

The findings of the present study suggest an association between prolonged smartphone use and self-reported fifth finger pain and repetitive strain symptoms among young adults. Although awareness of RSI was observed among some participants, important knowledge gaps remained regarding risk factors and preventive ergonomic practices. These findings highlight the importance of promoting ergonomic smartphone use, encouraging regular breaks during prolonged device use, and increasing awareness regarding preventive strategies to reduce the risk of fifth finger musculoskeletal symptoms.

## Introduction

Repetitive strain injury (RSI), colloquially referred to as “smartphone pinky,” is a musculoskeletal condition that has been associated with prolonged and repetitive smartphone use. Sustained support of a handheld device by the fifth finger may expose the finger to continuous mechanical loading, potentially resulting in pain, discomfort, numbness, tingling, and functional impairment [1].

During one-handed smartphone use, the fifth finger plays an important role in supporting the weight and maintaining the stability of the device. Prolonged mechanical loading of the fifth finger may increase stress on the small joints, tendons, ligaments, and surrounding soft tissues. Consequently, repetitive loading may contribute to pain, reduced grip strength, discomfort, and functional limitations of the hand [2].

Excessive smartphone use, particularly when combined with improper hand positioning and prolonged gripping, may contribute to inflammation of the tendon sheaths, muscular fatigue, and reduced pinch strength. Repeated exposure to these biomechanical stresses has been associated with musculoskeletal symptoms involving the hand and wrist [3].

Recent epidemiological studies have demonstrated a high prevalence of prolonged smartphone use among young adults. Daily smartphone use exceeding several hours has been associated with repetitive thumb and finger movements, sustained static postures, and an increased occurrence of upper-extremity musculoskeletal symptoms [1].

Smartphone use has become increasingly prevalent among young adults because of academic, occupational, and social demands. Activities such as texting, scrolling, gaming, and prolonged browsing require repetitive finger movements and sustained hand postures, which have been associated with musculoskeletal disorders affecting the upper extremity, particularly the hand and fingers [4,5].

RSI comprises a group of musculoskeletal disorders resulting from repetitive movements, prolonged static postures, excessive mechanical loading, and overuse of muscles, tendons, ligaments, and joints. Continuous smartphone use requires repetitive thumb and finger movements together with sustained gripping, which may increase stress on the small joints and soft tissues of the hand. Over time, these biomechanical demands may contribute to pain, stiffness, muscle fatigue, and functional limitations [6,7].

The fifth finger plays a significant role in supporting and stabilizing smartphones during one-handed use. Many users position the fifth finger beneath the device while simultaneously operating the touchscreen with the thumb of the same hand. Sustained pressure on the fifth finger resulting from the weight of the smartphone may contribute to repetitive microtrauma involving the muscles, tendons, and joints of the finger. Prolonged flexed positioning may further increase the risk of pain, discomfort, numbness, and fatigue involving the fifth finger and adjacent soft tissues [8].

Advances in smartphone technology have resulted in larger and heavier devices that frequently require prolonged gripping during daily use. Larger smartphones may increase mechanical loading on the supporting fingers, particularly during one-handed operation. In addition, improper holding patterns and poor hand posture may further increase stress on the hand and finger structures, thereby contributing to musculoskeletal discomfort [9].

Although numerous studies have investigated smartphone addiction and general musculoskeletal disorders, relatively few have specifically evaluated RSI involving the fifth finger and its association with smartphone overuse. A better understanding of this relationship may contribute to increased awareness and facilitate the development of preventive ergonomic strategies aimed at reducing hand-related musculoskeletal complications among young adults [6].

Therefore, the present study aimed to evaluate awareness of RSI involving the fifth finger and its association with fifth finger pain among young adults with smartphone overuse. In addition, the study investigated the relationship between smartphone usage characteristics, including duration of use, smartphone weight, and holding patterns, and self-reported fifth finger pain and discomfort [8].

Despite the increasing prevalence of smartphone use and the growing body of literature on smartphone-related musculoskeletal disorders, limited research has specifically investigated awareness of RSI involving the fifth finger and its association with fifth finger pain among young adults. Furthermore, awareness regarding appropriate ergonomic practices and preventive strategies remains insufficient. Therefore, evaluating awareness of this condition and its relationship with smartphone usage characteristics may provide valuable insights for developing educational interventions and promoting healthier smartphone use behaviors [10].

The present study aimed to evaluate awareness of RSI involving the fifth finger and its association with fifth finger pain among young adults with smartphone overuse. Additionally, the study sought to assess smartphone usage characteristics, including duration of smartphone use, device weight, and hand-holding patterns, and to examine their association with self-reported fifth finger pain. The findings of this study may contribute to improved awareness of smartphone-related musculoskeletal disorders and support the development of evidence-based ergonomic recommendations for injury prevention [11].

There is not much data available on the effect of overuse of smartphone handling in improper position. 

## Materials and methods

This cross-sectional observational study was conducted at the Department of Physiotherapy, Krishna College of Physiotherapy, Krishna Vishwa Vidyapeeth (Deemed to be University), Karad, Maharashtra, India, after obtaining approval from the Institutional Ethics Committee (Protocol No. KIMSDU/IEC/04/2026). Participant recruitment and data collection were carried out between 17 April 2026 and 24 April 2026. The study aimed to evaluate awareness of RSI involving the fifth finger and its association with fifth finger pain among young adults with smartphone overuse. A total of 188 participants meeting the predefined eligibility criteria were enrolled in the study. Male and female young adults who were regular smartphone users and provided informed consent were included. Individuals with a history of recent upper-limb trauma or injury, congenital abnormalities of the hand, previous hand surgery, severe neurological disorders, or any medical condition affecting hand function were excluded to minimize potential confounding factors.

Sample size calculation

The sample size was calculated using the single population proportion formula:

n = (Z² × p × q) / d², where n = required sample size; Z = standard normal deviate corresponding to the desired confidence level; p = expected proportion (prevalence) from previous literature; 1 − p (q) = complement of the expected proportion; d = absolute precision (margin of error).

Values used in the present study were confidence level = 85%; Z = 1.44; expected proportion (p) = 0.74 (74%); complement (q = 1 − p) = 0.26 (26%); absolute precision (d) = 0.05 (5%). Using these values, n = (1.44² × 0.74 × 0.26) / 0.05² = 160. Thus, the minimum required sample size was 160 participants. A total of 188 participants were finally included in the study, which exceeded the minimum required sample size.

Inclusion and exclusion criteria

The study included male and female young adults who were regular smartphone users and willing to participate by providing informed consent. Participants with recent upper limb trauma or injury, congenital abnormalities of the hand, previous hand surgery, severe neurological disorders, or any medical condition affecting hand function were excluded from the study. This ensured that the findings specifically reflected the association between smartphone overuse and fifth finger pain without interference from other underlying conditions.

Data collection procedure

Participants were recruited from Krishna College of Physiotherapy, Krishna Vishwa Vidyapeeth (Deemed to be University), Karad, as well as from the surrounding community. A structured Google Forms (Mountain View, CA, USA) questionnaire was distributed through WhatsApp and other social media platforms. Eligible young adults who met the inclusion criteria. The survey questionnaire was self-developed after an extensive review of the relevant literature on RSI, smartphone use, and fifth finger pain. The questionnaire was reviewed for content validity by a panel of 10 experts in physiotherapy and research methodology. Based on their suggestions, appropriate modifications were made to improve the clarity, relevance, and comprehensiveness of the questionnaire. A pilot study involving 10 participants was conducted to assess the feasibility, clarity, and comprehensibility of the questionnaire before the final data collection. The final questionnaire was converted into a Google Forms survey and distributed electronically through WhatsApp and other social media platforms (Appendix). Participants were invited to complete the questionnaire online using the survey link. Before accessing the questionnaire, all participants were provided with a participant information sheet

Data were collected using a semi-structured questionnaire containing demographic details, awareness regarding RSI, smartphone usage habits, and symptoms related to fifth finger pain.

Statistical analysis

The data collected through Google Forms were exported to Microsoft Excel (Redmond, WA, USA) for organization and analysis. Descriptive statistics, including frequencies and percentages, were used to summarize the participants' responses. The results are presented in the form of tables and pie charts.

Outcome measure

A self-structured questionnaire was developed as the primary outcome measure to assess awareness, symptoms, and smartphone usage patterns related to RSI of the fifth finger among young adults. The questionnaire consisted of three sections.

Section A: Awareness and Knowledge

This section assessed participants' knowledge of smartphone-related musculoskeletal problems, risk factors for fifth finger pain, and awareness of preventive and ergonomic strategies.

Section B: Symptoms and Attitudes

This section evaluated self-reported symptoms associated with smartphone overuse, including fifth finger pain, numbness, tingling, stiffness, fatigue, and discomfort. Participants' attitudes and perceptions regarding the effects of smartphone use on hand health were also assessed.

Section C: Smartphone Usage Patterns and Functional Limitations

This section examined smartphone usage duration, frequency of use, hand-gripping patterns, and difficulties in performing daily activities due to fifth finger pain. The responses were used to explore the relationship between smartphone overuse and functional impairment.

## Results

**Figure 1:**
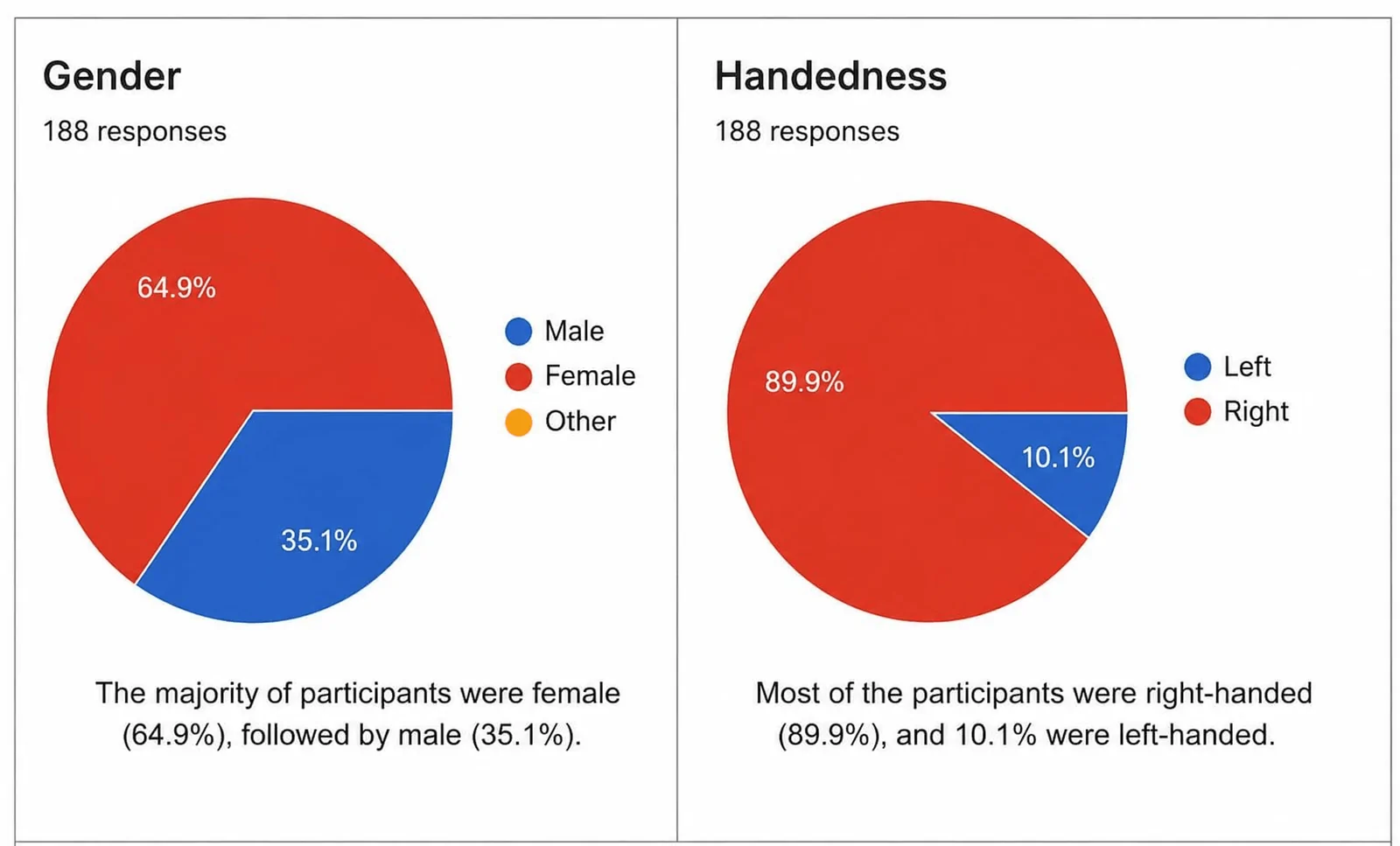
Distribution of participants by gender and handedness

Among the 188 participants, 66 (35.1%) were males and 122 (64.9%) were females. Most participants were right-handed (169; 89.9%), while 19 (10.1%) were left-handed. This indicates that the study population predominantly consisted of females and right-handed individuals (Figure 1).

Table 1 summarizes participants’ awareness regarding RSI of the fifth finger and associated risk factors. Among the 188 participants, 62 (33.0%) were well aware of RSI, whereas 45 (23.9%) were not aware of the condition. Regarding awareness of risk factors, 75 participants (39.9%) reported awareness of some risk factors, while 34 (18.1%) indicated awareness of most risk factors. A majority of participants, 99 (52.7%), recognized that prolonged smartphone use may contribute to fifth finger pain. Furthermore, 77 participants (41.0%) believed that maintaining proper smartphone holding techniques could help reduce the risk of RSI, and 97 (51.6%) acknowledged that taking frequent breaks may prevent repetitive strain. Regarding behavioral modification, 100 participants (53.2%) reported changing their smartphone holding position to reduce pain. Overall, these findings suggest a moderate level of awareness regarding RSI and its preventive strategies among the study participants.

**Table 1:** Participant awareness of repetitive strain injury and knowledge regarding smartphone use, associated risk factors and ergonomics prevention strategies

		Frequency	Percentage
Awareness of repetitive strain injury of the fifth finger due to smartphone overuse	Yes, well aware	62	33
	Not aware	45	23.9
	Heard but limited knowledge	61	32.4
	Heard but not sure	20	10.6
Awareness of risk factors associated with repetitive strain injury of the fifth finger due to smartphone overuse	Yes, aware of most risk factors	34	18.1
	Yes, aware of some risk factors	75	39.9
	No	51	27.1
	Not sure	28	14.9
Prolonged use of smartphone can affect 5^th^ finger	Definitely yes	56	29.8
	Probably yes	99	52.7
	No	13	6.9
	Not sure	20	10.6
Do you know causes of prolonged use of smartphones	Yes	90	47.9
	No	21	11.2
	Maybe	68	36.2
	Not sure	9	4.8
Are you aware about proper holding of smartphones	Yes	77	41
	No	42	22.3
	Maybe	51	27.1
	Not sure	18	9.6
Do you know that frequent breaks can prevent strain	Yes	97	51.6
	No	35	18.6
	Maybe	45	23.9
	Not sure	11	5.9
Have you changed your position of holding the phone because of pain	Yes, I changed it completely	45	23.9
	Yes, I changed it a little	100	53.2
	No, but I feel I should change	27	14.4
	No	16	8.5
	Total	188	100

Among the 188 participants, 28 (14.9%) reported frequently experiencing discomfort prior to smartphone use, 66 (35.1%) experienced discomfort occasionally, and 80 (42.6%) reported no discomfort. Regarding physical changes in the fifth finger, 21 participants (11.2%) reported clearly noticeable changes, while 56 (29.8%) reported mild but noticeable changes. A majority of participants believed that prolonged smartphone use could cause discomfort, with 86 (45.7%) responding "probably yes" and 58 (30.9%) responding "definitely yes." Furthermore, 113 participants (60.1%) agreed and 42 (22.3%) strongly agreed that pain in the fifth finger should be taken seriously. Additionally, 116 respondents (61.7%) agreed that changing hand position during smartphone use could help reduce pain (Table 2). Overall, these findings suggest that although a considerable proportion of participants reported experiencing physical discomfort or observable changes in the fifth finger, there was a strong consensus regarding the potential adverse effects of prolonged smartphone use, the importance of recognizing finger pain as a clinically relevant symptom, and the effectiveness of simple behavioral modifications, such as changing hand position, in alleviating discomfort.

**Table 2:** Distribution of self-reported physical symptoms, discomfort, and attitude regarding prolonged smartphone use among respondents.

		Frequency	Percentage
Have you felt any discomfort prior to smartphone	Yes, frequently	28	14.9
	Yes, occasionally	66	35.1
	No	80	42.6
	Not sure	14	7.4
Physical changes in 5^th^ finger	Yes, clearly noticeable	21	11.2
	Yes, mild change noticed	56	29.8
	No	89	47.3
	Not sure	22	11.7
Prolonged use of a smartphone can cause discomfort	Probably yes	86	45.7
	Definitely yes	58	30.9
	No	18	9.6
	Not sure	26	13.8
Perception of the seriousness of fifth finger pain due to smartphone use	Strongly agree	42	22.3
	Agree	113	60.1
	Not sure	32	17
	Disagree	1	0.5
Do you think changing the position of holding the smartphone helps to reduce strain on the 5th finger	Yes	116	61.7
	No	13	6.9
	Maybe	52	27.7
	Not sure	7	3.7
	Total	188	100

Among the 188 participants, 91 (48.4%) reported using their smartphones for two to four hours per day, while 52 (27.7%) used them for four to six hours daily. Regarding the duration of smartphone use associated with the onset of pain, 68 participants (36.2%) reported experiencing pain after less than two hours of use, whereas 56 (29.8%) reported pain after four to six hours of use. In terms of the overall duration of smartphone ownership, 68 participants (36.2%) had been using smartphones for three to five years. With respect to handling preference, the majority of respondents (127; 67.6%) predominantly used their right hand while operating their smartphones. Regarding handling habits, 87 participants (46.3%) reported that they sometimes supported their smartphones with the little finger, and 95 (50.5%) indicated that fifth finger pain sometimes affected their activities of daily living (ADLs). Additionally, 132 respondents (70.2%) reported feeling comfortable while using their smartphones. Most participants (97; 51.6%) used smartphones weighing between 170 and 200 g (Table 3). Overall, these findings suggest that prolonged daily smartphone use, specific handling behaviors, and the use of relatively heavier devices may contribute to localized fifth finger discomfort and mild limitations in ADLs.

**Table 3:** shows distribution of daily smartphone exposure, hand gripping behaviour and functional limitation among respondents. ADLs: activities of daily living

		Frequency	Percentage
Daily use of smartphone	<2	23	12.2
	2-4 hours	91	48.4
	4-6 hours	52	27.7
	>6	22	11.7
	Total	188	100
Duration of smartphone that causes pain	<2	68	36.2
	2-4 hours	36	19.1
	4-6 hours	56	29.8
	>6	28	14.9
How many years have you used a smartphone	<1	19	10.1
	1-3 years	46	24.5
	3-5 years	68	36.2
	>5	55	29.3
Which hand gets more use during handling	Left	20	10.6
	Right	127	67.6
	Both	37	19.7
	Not sure	4	2.1
Do you support your phone with little finger	Never	24	12.8
	Sometimes	87	46.3
	Often	38	20.2
	Always	39	20.7
Does 5^th^ finger pain affect ADL	Never	65	34.6
	Sometimes	95	50.5
	Often	18	9.6
	Always	10	5.3
How comfortable is your phone	Very comfortable	31	16.5
	Comfortable	132	70.2
	Uncomfortable	19	10.1
	Very uncomfortable	6	3.2
Weight of smartphone	<170gm	36	19.1
	170-200gm	97	51.6
	200-230gm	45	23.9
	>230gm	10	5.3
	Total	188	100

## Discussion

The present study aimed to evaluate the awareness of RSI of the fifth finger and its association with fifth finger pain among young adults in relation to smartphone overuse. The findings indicated an association between prolonged smartphone use, higher smartphone weight, and improper smartphone handling patterns with the occurrence of fifth finger pain and discomfort among the participants. Young adults commonly use smartphones for communication, gaming, social networking, and educational activities, often for extended periods without adequate rest. Prolonged repetitive finger movements and sustained gripping postures during smartphone use may increase mechanical stress on the muscles, tendons, and joints of the hand and fingers, thereby contributing to the development of RSI [4,5].

In the present study, a considerable proportion of participants reported prolonged daily smartphone use. Extended smartphone use involves repetitive finger movements, including texting, scrolling, typing, and gaming, as well as sustained hand postures, which may increase mechanical loading on the small joints, muscles, tendons, and other soft tissues of the hand. Repetitive movements performed over prolonged periods with inadequate recovery may contribute to muscle fatigue, pain, and discomfort. These findings are consistent with those reported by Berolo et al., who demonstrated that prolonged handheld device use is associated with musculoskeletal symptoms among smartphone users [4].

The findings of the present study also suggested an association between smartphone weight and fifth finger discomfort. Larger and heavier smartphones may require greater support from the hand and fingers, particularly during single-handed use. A common handling pattern involves supporting the smartphone with the fifth finger while operating the device with the thumb, which may increase mechanical loading on the little finger. Prolonged exposure to this loading may contribute to pain, strain, and fatigue in the fifth finger. These findings are consistent with previous studies reporting that prolonged smartphone use and suboptimal hand postures are associated with an increased prevalence of upper extremity musculoskeletal symptoms among young adults [6,7].

Another important finding of the present study was the observed association between smartphone holding patterns and fifth finger pain. Improper hand positioning and prolonged gripping postures may increase mechanical stress on the fifth finger and surrounding anatomical structures. Sustained flexion of the fifth finger while supporting the smartphone may contribute to repetitive microtrauma and discomfort. Repeated exposure to these mechanical stresses over time may increase the risk of developing RSI and functional impairment. These findings are consistent with previous studies reporting an association between smartphone use, repetitive finger movements, prolonged gripping postures, and hand and finger pain [7,8].

The findings of the present study highlight the potential importance of preventive strategies for reducing the risk of RSIs associated with smartphone use. Ergonomic interventions, including limiting prolonged continuous smartphone use, taking regular breaks, varying smartphone holding patterns, alternating between the right and left hands during device operation, and maintaining appropriate hand posture, may help reduce mechanical stress on the fifth finger and surrounding anatomical structures. Promoting awareness of ergonomic practices and encouraging early behavioral modifications may contribute to minimizing the risk of long-term musculoskeletal complications associated with excessive smartphone use [6].

The present study has several limitations that should be considered when interpreting the findings. Data were collected using a self-administered Google Forms questionnaire, and therefore relied on participants' self-reported responses, which may be subject to recall and reporting bias. The study sample was limited to students and young adults, which may restrict the generalizability of the findings to other age groups and populations. Furthermore, no objective clinical examination or physical assessment was performed to confirm the presence or severity of fifth finger pain or RSI. Future research should include larger and more diverse study populations, along with objective clinical assessment methods, to further investigate the relationship between smartphone overuse and fifth finger pain [8].

Overall, the findings of the present study suggest that prolonged smartphone use, greater smartphone weight, and improper smartphone holding patterns are associated with an increased occurrence of fifth finger pain and may contribute to the development of RSI among young adults. These findings underscore the potential importance of promoting awareness of appropriate smartphone usage habits and ergonomic practices to reduce mechanical stress on the hand and minimize the risk of musculoskeletal disorders associated with smartphone use [4].

The study conducted by Banadaki et al. (2024) reported a significant association between prolonged smartphone use and the occurrence of hand pain among college students. The authors found that extended smartphone use, particularly for activities such as texting, social media browsing, and academic tasks, was associated with an increased prevalence of musculoskeletal discomfort involving the muscles, tendons, and joints of the hands and fingers. Participants with longer daily smartphone use were more likely to report pain, stiffness, and fatigue in the hands. The study suggested that repetitive thumb and finger movements, combined with prolonged device handling, may increase mechanical stress on the musculoskeletal structures of the hand. Furthermore, the authors identified poor hand posture during smartphone use as an important factor associated with hand discomfort, with sustained non-neutral hand positions being linked to a higher prevalence of musculoskeletal symptoms. They emphasized the importance of considering hand posture, device size, and duration of smartphone use when addressing smartphone-related musculoskeletal disorders. These findings further support existing evidence that excessive smartphone use is associated with adverse upper limb musculoskeletal outcomes and highlight the potential benefits of ergonomic practices, regular breaks, and limiting prolonged smartphone use to reduce the risk of hand pain and discomfort among young adults [10].

Ahmed et al. (2022) investigated the relationship between smartphone addiction and musculoskeletal pain among college-going students and reported a significant association between excessive smartphone use and pain in the neck, shoulder, elbow, and hand regions. The study demonstrated that students with higher levels of smartphone addiction experienced a greater prevalence and severity of musculoskeletal discomfort. Prolonged smartphone use involves repetitive hand and finger movements along with sustained static postures, which may increase mechanical stress on the musculoskeletal structures and contribute to pain and fatigue in the upper extremities. The findings indicated that increased dependence on smartphones may negatively influence musculoskeletal health and daily functional activities. Furthermore, the authors highlighted that extended smartphone use may promote poor postural behaviors, including forward head posture and prolonged upper limb positioning, which can increase stress on the cervical spine and surrounding musculature. The observed association between smartphone addiction and upper limb pain emphasizes the importance of ergonomic awareness, regular rest intervals, and responsible smartphone usage practices. Preventive strategies aimed at reducing excessive smartphone use and promoting healthy postural habits may help minimize the risk of musculoskeletal disorders among young adults. These findings contribute to the growing evidence that repetitive and prolonged use of handheld devices may be associated with discomfort and functional limitations in the neck, shoulder, elbow, and hand regions [11].

The findings of the present study are supported by Demirkıran et al. (2024), who investigated the effects of smartphone use on morphological and radiological changes in the fifth finger. Their study demonstrated that prolonged smartphone use may be associated with structural alterations in the little finger due to repetitive loading and sustained gripping postures during device handling. The authors further reported that larger and heavier smartphones, along with longer daily usage durations, were associated with more pronounced changes in fifth-finger morphology. These findings suggest that continuous smartphone handling may increase mechanical stress on the hand and fingers, potentially contributing to discomfort and musculoskeletal adaptations. The results highlight the importance of adopting ergonomic smartphone usage practices and limiting prolonged device use to reduce the risk of hand-related musculoskeletal problems [12].

Depreli and Angin reported that smartphone addiction and improper smartphone usage postures were associated with increased hand pain and reduced hand function. Prolonged smartphone use in non-neutral positions may increase mechanical stress on the muscles, tendons, and other soft tissues of the hand and wrist. These findings are consistent with the present study, suggesting that excessive smartphone use and inappropriate handling patterns may negatively influence hand health and functional performance among young adults [13].

Athar et al. (2025) reported that excessive smartphone use is associated with impaired hand function and an increased prevalence of hand-related disorders. The authors found that prolonged smartphone use may contribute to pain, discomfort, reduced grip efficiency, and increased musculoskeletal strain within the hand. These findings support the results of the present study and highlight the potential adverse effects of excessive smartphone use on hand health and functional performance. The study emphasizes the importance of increasing awareness regarding safe smartphone usage practices to reduce the risk of hand-related musculoskeletal problems [14].

Varmazyar (2026) reported that prolonged smartphone use is significantly associated with hand pain among university students. The review identified factors such as extended daily smartphone usage, repetitive thumb movements, and inappropriate hand postures as important contributors to hand discomfort. These findings support the present study by suggesting that intensive smartphone use may increase the risk of hand pain and functional limitations. The study emphasizes the importance of adopting ergonomic smartphone usage practices and incorporating regular breaks to reduce the risk of hand-related musculoskeletal problems [15].

This study has several limitations that should be considered when interpreting the findings. First, the cross-sectional study design limits the ability to establish a causal relationship between smartphone overuse and fifth finger pain. Second, the data were obtained through self-reported responses using an online questionnaire, which may be influenced by recall bias and response bias. Third, participants were recruited through convenience sampling from Krishna College of Physiotherapy and the surrounding community, which may restrict the generalizability of the findings to the wider population.

## Conclusions

The present study concludes that awareness regarding RSI of the fifth finger among young adults is moderate but remains incomplete. Although many participants recognized that prolonged smartphone use may contribute to fifth finger pain and discomfort, a considerable proportion demonstrated insufficient knowledge regarding associated risk factors, appropriate smartphone handling techniques, and preventive strategies. The majority of respondents reported using smartphones for several hours daily and frequently supported their devices with the fifth finger, which may increase mechanical stress and strain on the finger.

Furthermore, the findings indicate an association between excessive smartphone use and the occurrence of fifth finger pain, discomfort, and functional limitations in daily activities. A considerable number of participants reported experiencing discomfort, observing physical changes in the fifth finger, and acknowledging that modifications in smartphone holding positions and regular breaks may help reduce symptoms. These findings emphasize the importance of improving awareness and providing education regarding ergonomic smartphone usage practices and preventive measures to minimize the risk of RSI and fifth finger pain among young adults.

## Appendices

Questionnaire

Section A: Demographic Data

Name-

Age-

Gender-

Occupation-

Handedness-

Section B: Knowledge

1. Are you aware of repetitive strain injury of fifth finger? [Deformity]

a. Yes, well aware

b. Not aware

c. Heard but limited knowledge

d. Heard but not sure

2. Are you aware of risk factors causing repetitive strain injury of the fifth finger?

a. Yes, aware of most risk factors

b. Yes, aware of some risk factors

c. No

d. Not sure

3. Do you think prolonged use of a smartphone can affect the 5th finger of the hand?

a. Definitely yes

b. Probably yes

c. No

d. Not sure

4. Do you know that prolonged use of a smartphone may cause: indentation (arch), numbness, tingling in 5th finger?

a. Yes

b. No

c. Not sure

d. Maybe

5. Are you aware of the proper way of holding a smartphone?

a. Yes

b. No

c. Not sure

d. Maybe

6. Do you know that frequent breaks from using a smartphone can help to prevent strain on the 5th finger?          

a. Yes

b. No

c. Not sure

d. Maybe

7. Have you changed your position of holding the phone because of pain?

a. Yes, I changed it completely

b. Yes, I changed it a little

c. No, but I feel I should change

d. No, I have not changed

Section C: Attitude

1. Have you felt any discomfort prior to smartphone use?

a. Yes, frequently

b. Yes, occasionally

c. No

d. Not sure

2. Have you observed any arch at your 5th finger?

a. Yes, clearly noticeable.

b. Yes, mild change noticed

c. No

d. Not Sure

3. Do you think prolonged use of smartphone can cause discomfort in your 5th finger?

a. Probably yes

b. Definitely yes

c. No

d. Not sure

4. Pain in 5th finger should be taken seriously among young adults?

a. Strongly agree

b. Agree

c. Not sure

d. Disagree

5. Do you think changing the position of holding the smartphone helps to reduce strain on the 5th finger?

a. Yes

b. No

c. Not sure

d. Maybe

Section D: Practice

1. How often do you use your smartphone daily?

a. <2 hours

b. 2-4 hours

c. 4-6 hours

d. >6 hours

2. What duration of continuous use of a smartphone may lead to the pain?

a. <2 hours

b. 2-4 hours

c. 4-6 hours

d. >6 hours

3. For how many years have you been using a smartphone?

a. <1 year

b. 1-3 years

c. 3-5 years

d. >5 years

4. Which hand do you use more while using smartphone?

a. Left

b. Right

c. Both

d. Not sure

5. Do you support the phone with your little finger while holding it?

a. Never

b. Sometimes

c. Often

d. Always

6. Does little finger pain affect activities of daily living? Ex. typing, writing, gripping

a. Never

b. Sometimes

c. Often

d. Always

7. How comfortable is your phone size during prolonged use?

a. Very comfortable

b. Comfortable

c. Uncomfortable

d. Very uncomfortable

8. Approximate weight of your phone?

a. <170 gm

b. 170-200 gm

c. 200-230 gm

d. >230 gm

## References

[1] Aldanyowi SN, Al Haboob LM, Al Mssallem NI (2024). The hidden pain: understanding smartphone pinky awareness and risk perception in the eastern province population. Cureus.

[2] Ayyildiz A, Inceoglu SC (2025). Smartphone pinky: myth or reality. Med Bull Haseki.

[3] Al-Barashdi H, Bouazza A, Jabur N (2015). Smartphone addiction among university undergraduates: a literature review. J Sci Res Rep.

[4] Berolo S, Wells RP, Amick BC 3rd (2011). Musculoskeletal symptoms among mobile hand-held device users and their relationship to device use: a preliminary study in a Canadian university population. Appl Ergon.

[5] Gustafsson E, Thomée S, Grimby-Ekman A, Hagberg M (2017). Texting on mobile phones and musculoskeletal disorders in young adults: a five-year cohort study. Appl Ergon.

[6] Sharan D, Mohandoss M, Ranganathan R, Jose J (2014). Musculoskeletal disorders of the upper extremities due to extensive usage of hand held devices. Ann Occup Environ Med.

[7] Kim HJ, Kim JS (2015). The relationship between smartphone use and subjective musculoskeletal symptoms and university students. J Phys Ther Sci.

[8] Alsalameh AM, Harisi MJ, Alduayji MA, Almutham AA, Mahmood FM (2019). Evaluating the relationship between smartphone addiction/overuse and musculoskeletal pain among medical students at Qassim University. J Family Med Prim Care.

[9] Eitivipart AC, Viriyarojanakul S, Redhead L (2018). Musculoskeletal disorder and pain associated with smartphone use: a systematic review of biomechanical evidence. Hong Kong Physiother J.

[10] Banadaki FD, Rahimian B, Moraveji F, Varmazyar S (2024). The impact of smartphone use duration and posture on the prevalence of hand pain among college students. BMC Musculoskelet Disord.

[11] Ahmed S, Mishra A, Akter R (2022). Smartphone addiction and its impact on musculoskeletal pain in neck, shoulder, elbow, and hand among college-going students: a cross-sectional study. Bull Fac Phys Ther.

[12] Demirkıran ND, Ozmanevra R, Oner SK, Kozlu S, Dülgeroglu T (2024). An investigation on the effect of smartphone use on morphological and radiological changes of the fifth finger. J Contemp Med.

[13] Depreli O, Angin E (2024). The relationship between smartphone usage position, pain, smartphone addiction, and hand function. J Back Musculoskelet Rehabil.

[14] Athar M, Hashmi J, Saleem MM, Azam J, Umar SA, Mahmmoud Fadelallah Eljack M (2025). Impact of excessive phone usage on hand functions and incidence of hand disorders. Ann Med Surg (Lond).

[15] Varmazyar S (2026). Smartphone use and related factors with hand pain among university students: a systematic review. BMC Musculoskelet Disord.

